# Supplementary material for: Mouse models of human PIK3CA-related brain overgrowth have acutely treatable epilepsy
Source: eLife. 2015 Dec 3;4:e12703. doi: 10.7554/eLife.12703 (PMC4744197; doi:10.7554/eLife.12703)
Supplement: Supplementary file 1. — Table shows the list of parameters customized in the stimulator panel dialog box of LabChart 8 Software, in order to randomize the speed and direction of rotation of the motor used for the sleep deprivation study. The stimulation program cycle, comprising segments 1–12, was repeated through the entire duration of the sleep deprivation experiment (i.e. for 5 hr). DOI: http://dx.doi.org/10.7554/eLife.12703.024 [file elife-12703-supp1.doc]

| **Segment Number** | **1** | **2** | **3** | **4** | **5** | **6** | **7** | **8** | **9** | **10** | **11** | **12** |
| --- | --- | --- | --- | --- | --- | --- | --- | --- | --- | --- | --- | --- |
| **Segment Type** | **Triangle** | **Sine** | **Delay** | **Biphasic Pulse** | **Step** | **Triangular Pulse** | **Step** | **Biphasic Pulse** | **Triangle** | **Sine** | **Delay** | **Delay** |
| **Cycles/ Repeats** | 7 | 8 | - | 8 | 1 | 4 | 1 | 1 | 5 | 6 | - | - |
| **Frequency (Hz)** | 1 | 1 | - | - | - | - | - | - | 1 | 1 | - | - |
| **Amplitude (V)** | 0.8 | 0.8 | - | - | - | - | - | - | 0.8 | 0.8 | - | - |
| **Pulse Height (V)** | - | - | - | 1 | - | 0.8 | - | 0.8 | - | - | - | - |
| **Pulse Width (sec)** | - | - | - | 1 | - | 4 | 0.1 | 10 | - | - | - | - |
| **Skew** | 0 | - | - | - | - | - | - | - | 0 | - | - | - |
| **Step number** | - | - | - | - | 3 | - | 3 | - | - | - | - | - |
| **Time (sec)** | - | - | 10 | - | - | - | - | - | - | - | 10 | 10 |
| **Step Pulse Initial Height (V)** | - | - | - | - | 0 | - | 0 | - | - | - | - | - |
| **Step Pulse Final Height (V)** | - | - | - | - | 1 | - | 0.8 | - | - | - | - | - |
| **Step Width (sec)** | - | - | - | - | 5 | - | 0.2 | - | - | - | - | - |
| **End Delay (sec)** | - | - | - | 0 | - | 0 | - | 0 | - | - | - | - |

The stimulation program cycle, comprising segments 1-12, was repeated through the entire duration of the sleep deprivation experiment (i.e. for 5 hours).

**Supplementary file 1: Parameters for motor rotation in LabChart 8 Software**
